# High-Resolution Melting Analysis as an Appropriate Method to Differentiate between *Fasciola hepatica* and *F. gigantica*

**Published:** 2019-03

**Authors:** Ahmad HOSSEINI-SAFA, Mohammad Bagher ROKNI, Sayed Hussain MOSAWI, Peyman HEYDARIAN, Hakim AZIZI, Afshin DAVARI, Mojgan ARYAIEPOUR

**Affiliations:** 1. Department of Medical Parasitology and Mycology, School of Public Health, Tehran University of Medical Sciences, Tehran, Iran; 2. Department of Biology and Microbiology, Faculty of Medical Technology, Khatam Al-Nabieen University, Kabul, Afghanistan; 3. Department of Medical Parasitology and Mycology, School of Medicine, Qazvin University of Medical Sciences, Qazvin, Iran; 4. Department of Microbiology and Parasitology, School of Medicine, Zabol University of Medical Sciences, Zabol, Iran; 5. Department of Medical Parasitology and Mycology, Shahid Beheshti University of Medical Sciences, Tehran, Iran

**Keywords:** High-resolution melting (HRM), *Fasciola hepatica*, *Fasciola gigantica*, *CoxI*

## Abstract

**Background:**

Fasciolosis is a shared disease between humans and livestock caused by hepatic trematodes; *Fasciola hepatica* and *F. gigantica*. Differentiate between the two species of this genus is essential. High-Resolution Melting (HRM) Analysis represents a new approach to this issue. This method can be performed right after termination of Real-Time PCR. This technique has not been used for identification of adult *F. hepatica* and *F. gigantica* genotypes. The aim of this study was to determine *Fasciola* species by using HRM in isolates taken from Iran, respectively.

**Methods:**

Ninety-three *Fasciola* spp. samples were collected from infected slaughtered animals in different regions of Iran, including North West (Ardebil Province) and South East (Zahedan Province) during 2016. Genomic DNA from the samples was extracted using a DNA extraction kit and then after Real-Time PCR amplification, HRM was done.

**Results:**

Overall, 59 and 34 isolates were identified as *F. hepatica* and *F. gigantica*, respectively. The percentages of each species from animals were as follows: sheep (*F. hepatica*, 80.39% and *F. gigantica*, 19.61%), cattle (*F. hepatica*, 42.85% and *F. gigantica*, 57.15%).

**Conclusion:**

HRM technique developed in the present study is a powerful, rapid and sensitive technique for epidemiological survey and molecular identification between *F. hepatica* and *F. gigantica*.

## Introduction

The common liver flukes *Fasciola hepatica* and *F. gigantica* are causative agents of fasciolosis: a global zoonotic parasitic disease. Although fasciolosis commonly occurs in liver and biliary ducts, ec-topic fasciolosis may occur in the peritoneal cavity, lungs, subcutaneous tissue, lymph nodes, eye, and other locations (1, 2). Besides causing disease in humans, it can induce mortality and morbidity in sheep and cattle industry (3).

As these flat worms parasitize mollusks and vertebrate hosts, accurate and fast detection of them is of vital importance for facilitating control and prevention strategies of fasciolosis in humans and animals (4). One of the fast, sensitive and specific approach for detection and evolutionary analysis of *Fasciola* spp. are molecular techniques. Despite existence of many diagnosis tools like microscopy, radiology, ultrasound, CT, MRI, serological and clinical tests for detection of fasciolosis, unsatisfactory amounts of sensitivity and specificity have been obtained in many studies (5). Emerging of molecular methods was a turning point to accurate and precise detection and species identification of parasites. Molecular methods not only offer an appropriate impediment for detection of *Fasciola* spp. in mammals, but also in detection of infected snails in the epizootiological studies of these flukes (6).

Toward this end, many molecular techniques have been used by researchers that vary from conventional PCR methods like PCR-RFLP, PCR-SSCP and multiplex PCR to quantitative and qualitative real-time PCR (qRT-PCR) technology (7–10).

Indeed real-time PCR has brought the detection of parasitic diseases to another level, with significant sensitivity, easier performance, and no post-PCR operation compared to conventional PCR methods. HRM analysis is a novel method that allows us rapid screening and detection of closely related species in a laboratory (11). The steps in HRM analysis entangle amplification of the wanted region in the presence of a specialized dsDNA binding dye and step by step denaturation of amplicons by increasing the temperature in small increments in order to produce a characteristic melting profile called melting analysis (12). To date, the HRM has mostly been used in molecular studies of parasitic protozoa and rarely for parasitic worms (11,13,14).

The genes targeted for molecular strategies against *Fasciola* spp. are the *ITSI* and *ITSII* of nuclear DNA and *COI* from mitochondrial DNA. The *COI* gene fragment introduced as a most variable marker recommended for future analyses (15,3).

In this study, we developed a qualitative real-time PCR method appeared with HRM analysis for the detection of the two *Fasciola* spp. In samples taken from Ardebil and Zahedan provinces, northwestern and southern Iran, respectively.

The study was conducted as a complementary case to previous studies on genotype (16) and morphological verifications (17).

In this study, we aimed to develop a qualitative real-time PCR method appeared with HRM analysis for the detection of the two *Fasciola* spp. Samples were taken from Ardebil and Zahedan provinces, northwestern and southern Iran, respectively.

## Material and Methods

### Sample collection

**Fig. 1:**
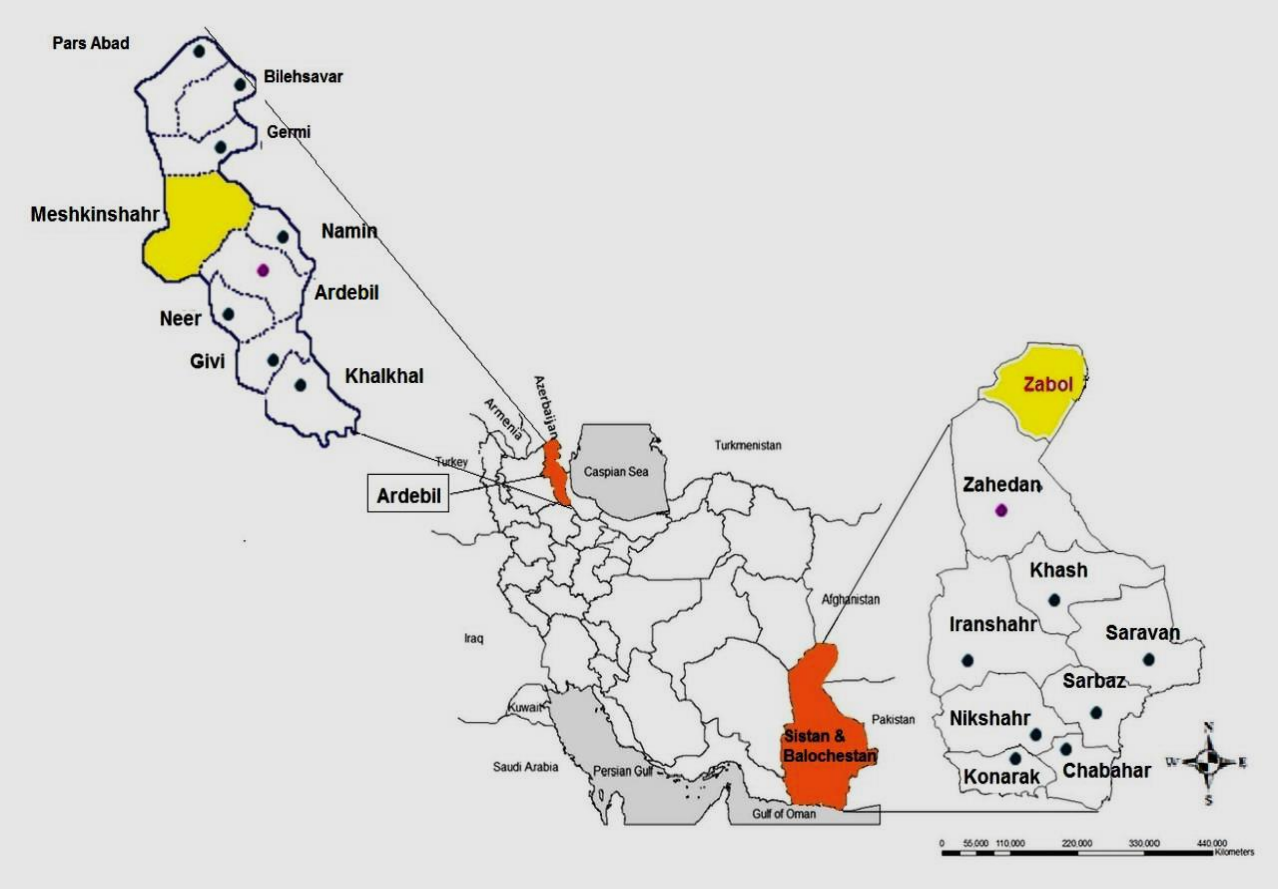
Sistan and Baluchestan Province (South East) and Ardebil Province (North West), Iran. The Meshkinshahr and Zabol cities are highlighted in yellow

Ninety-three adult trematodes of *Fasciola* spp. located within the bile ducts of infected cattle (n=42 samples) and sheep (n=51 samples), were collected from slaughterhouses in two different regions of Iran from Nov 2016 to Jan 2017. The study was conducted in Meshkinshahr City, Ardebil Province, North West and Zabol City, Sistan, and Baluchistan Province, South East, Iran (Fig. 1). The flatworms were extensively washed in physiological saline and preserved in >70° (v/v) ethanol and then frozen at −20 °C until DNA extraction.

Ethics Committee of Tehran University of Medical Sciences approved the study.

### DNA extraction

Before DNA extraction, all individual worms rinsed three times with PBS to remove the ethanol. Genomic DNA (Gdna) was extracted from small portion of apical region of adult trematodes to avoid entry female genitalia that likely to outer sperm. Gdna from individual worms was extracted using a QIAamp DNAeasy, Hilden, Germany, according to the manufacturer’s reference protocols with some modifications. The concentration of the extracted DNA was specified by NanoDrop (Thermo Scientific, Rockford, IL, USA), and after that, the samples were stored at −20 °C for further analysis.

### DNA amplification and HRM

The mitochondrial sequences were amplified using specific primers for the COI gene, (forward A.H.S-CO1-F 5′- GGGCATCCTGAGGTTTATGT-3′ and reverse A.H.S-CO1-R 5′- AACATTATCAACCAGGAAAAGACC-3′ design was carried out using the sequences available on GenBank and in continuation. Primers were obtained using the program PRIMER BLAST (http://www.ncbi.nlm.nih.gov/tools/primer-blast/) and rechecked with Beacon Designer8.12, PREMIER BIOSOFT software from the consensus sequence obtained by the multiple alignments, with an expected amplicon size of 266 bp for all the sequences.

PCR was performed in 20 Ml final reaction volume containing 10 Ml master mix (Type-it HRM PCR Kit; Qiagen, Hilden, Germany), 5.2 Ml distilled water, 0.4 Ml of each primer, and 4 Ml of template DNA. Enzymatic reaction was performed as follows: the reaction mixture was heated for initial denaturation step at 95 °C for 10 min, followed by 40 cycles of amplification performed at 95 °C for 30 sec for denaturation step, 55 °C for 40 sec, for annealing section, 72 °C for 30 sec related to extension portion, and a final extension step at 72 °C for 6 min after 40 cycles. HRM temperature was raised from 70 °C to 95 °C. During this process, the amplicons obtained from PCR were denatured prior to the development of melting curves in the inflexion point where changes in fluorescence with respect to changes in temperature (Df/Dt) were recorded with a ramp of 0.3 °C/sec (18). Fluorescence dye signaling was measured after each cycle. The kit contained the novel double-stranded DNA-binding fluorescent dye, EvaGreen, and an optimized HRM PCR master mix buffer, consisting of HotStarTaq plus DNA polymerase, Q-Solution, and dNTPs.

We used a positive standard control for *F. hepatica* and *F. gigantica* available in the Department of Medical Parasitology and Mycology, School of Public Health, Tehran University of Medical Sciences.

Real-time PCR was carried out in a Mini Opticon real-time PCR detection system (Applied Biosystems Step One Plus Inc., CA, USA). The Real-Time amplification result and T_m_ analysis were obtained using the Step One Plus^TM^ software ver. 2.3 (Life technologies^@^). T_m_ analysis was repeated three times in each run to confirm the repeatability of the T_m_ assay by estimating the T_m_ variation within a PCR amplification (intra-assay), and between PCR amplifications (inter-assay). The coefficient of variation (CV) was calculated by dividing the standard deviation (SD) by the arithmetic mean of the measured values of Tm (CV = SD[Le, 2012 #11]/mean value).

Furthermore, to check the uniformity of temperature in the cycler block, a number of samples were re-amplified at different positions of the cycler block during the same amplification cycle. The intra-assay CVs represent the mean CVs of the results obtained from the replications of *Fasciola* spp.

## Results

### Real-Time PCR amplification and HRM analysis

In accordance with Real-Time PCR and HRM analysis procedure, among 93 samples containing cattle (n=42 samples) and sheep (n=51 samples) were amplified using partial sequence of *COI* gene of *Fasciola* spp.*,* and then HRM was performed. T_m_ analysis was repeated three times in each run to confirm the repeatability of the T_m_ assay (Table 1).

**Table 1:** Mean T_m_, SD, and CV calculated based on intra- and inter-assay of *cox1* gene sequence of *Fasciola* spp.

***Gene***	***Mean Tm (°C)***	***SD***	***Intra-assay CV (%)***	***Inter-assay CV (%)***
*cox1*				
*F. hepatica*	76°C	0.14	0.05	0.12
*F. gigantica*	77.1°C	0.10	0.07	0.11

**Fig. 2:**
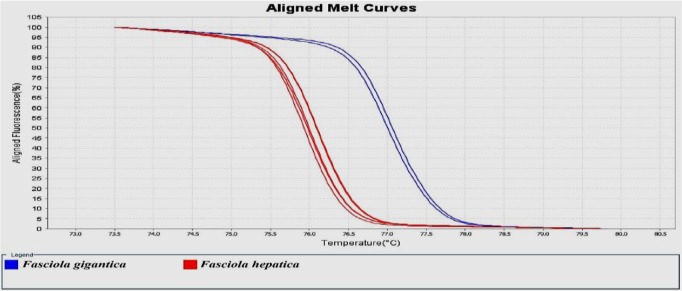
HRM based on (EvaGreen) Aligned Melt curves analyses and identified *Fasciola* spp. In sheep and cattle using *COI* gene

**Fig. 3:**
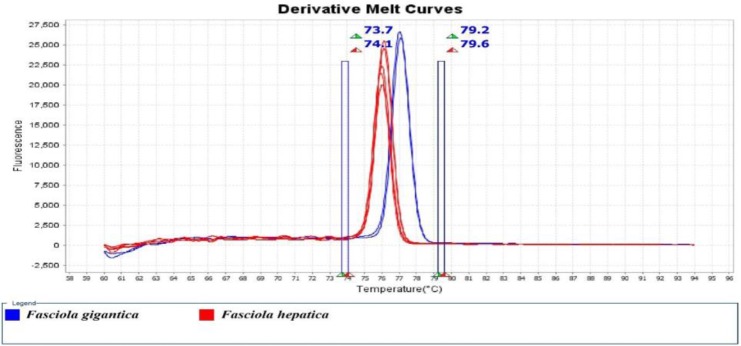
Derivative Melt Curves of the analyzed and identified *Fasciola spp.* In sheep and cattle using *COI* gene

The result of HRM analysis showed that 59 and 34 isolates were identified as *F. hepatica* and *F. gigantica*, respectively. The percentages of each species from animals were as follows: Sheep (*F. hepatica*, 80.39% and *F. gigantica*, 19.61%), cattle (*F. hepatica*, 42.85% and *F. gigantica*, 57.15%). The melting curve and HRM curve analysis of the *Fasciola* spp. Identified and shown in Figs. 2 and 3. The real-time PCR melting curve results indicated that the mean Tm, SD, CV calculated based on intra- and inter-assay was separated by each genotype of *Fasciola* spp.

## Discussion

Following previous studies and because of the importance of fascioliasis in Iran, more comprehensive studies on this parasite and the diagnosis of its species are necessary to achieve this goal (19, 20). In the previous studies, PCR-RFLP technique on *ITSI* gene was used to determine the species of *Fasciola* and compare it with the sequencing and morphology methods (16,17).

In the present assay, we successfully developed a real-time PCR and HRM technique on COI gene for rapid, sensitive and precise differentiation of *F. hepatica* and *F. gigantica* using only a single pair of primers. The average T_m_ variation obtained by melting curve analysis was about 1°C. This finding indicates a sufficient ability and reliability of the assay for distinguishing these two parasites.

The real-time PCR and HRM assay often are able to scan even a one SNP, it is likely to be able in significant distinct of the intermediate form of *Fasciola* spp. in endemic regions. However, this method cannot be a proper substitute for sequencing in terms of aforementioned point.

The results of this study were completely consistent with the previous studies carried out using the PCR-RFLP technique on *ITSI* gene, sequencing and morphology methods on the *Fasciola*. isolates of Ardabil Province (16, 17).

Despite the satisfactory results of molecular methods, according to previous studies different sensitivity and specificity have been acquired from variety of DNA based techniques (from conventional to real-time PCR) (15, 21, 22). There are many target genes employed for detection of *Fasciola* spp. including rRNA gene (*ITSI* and *ITSII*) of genomic DNA (22), and NADH and COI of the mitochondrial DNA (17, 19, 23). Several PCRRFLP assays have been described for discrimination of *F. hepatica, F. gigantica* that targeting the 28S rRNA, *ITSI* and *ITSII* (15, 16, 18, 21). In addition, a PCR-SSCP on *ITSI* region of rRNA was defined for distinction between *F. hepatica, F. gigantica* and the intermediate species in China (7). A multiplex PCR was designed for simultaneous amplification of mitochondrial DNA and ITS region of *Fasciola* spp. in mammalian and intermediate hosts (24, 25). Real-time PCR, as a more sensitive molecular approach, has been used in some studies focused on liver fluke (7, 10).

Whenever HRM is incorporated in real-time PCR, this allows us nonstop quantitative detection of the parasites. The present study has been done for the first time in the world while there are some studies used HRM for identification and genotyping of other parasites. For example, the real-time PCR and HRM technique was used for identification of *Echinococcus* spp. in Isfahan Province of Iran (26, 27). In addition this technique was applied for genotyping of protozoan parasites like *Plasmodium* spp., *Giardia* spp. and *Cryptosporidium* spp. (4, 7, 12, 28).

It has been described as a robust assay for the rapid detection for phylum Platyhelminthes such as *Fascioloides magna*, *Schistosoma* spp., *Clonorchis sinensis* and *Opisthorchis viverrini* (6, 29).

Finally, this assay needs to be more developed by performing on large sample size in wide geographical regions in the world especially in endemic areas such as Iran. Besides, the efficacy of this technique should be evaluated on discrimination of intermediate species in the region that both of *F. hepatica* and *F. gigantica* exist.

The limitations of this study included the small sample size and non-overlapping all geographic regions of Iran. The advantage, however, is that HRM technique was performed for the first time in the world to molecular identification between *F. hepatica* and *F. gigantica.*

## Conclusion

HRM technique developed in present study is a powerful, rapid and sensitive technique for epidemiological survey and molecular identification between *F. hepatica* and *F. gigantica*.

## Ethical considerations

Ethical issues (Including plagiarism, informed consent, misconduct, data fabrication and/or falsification, double publication and/or submission, redundancy, etc.) have been completely observed by the authors.
